# Source Apportionment of Potentially Toxic Elements in Agricultural Soils of Yingtan City, Jiangxi Province, China: A Principal Component Analysis–Positive Matrix Factorization Method

**DOI:** 10.3390/toxics13040267

**Published:** 2025-03-31

**Authors:** Shaoting Chen, Hongmei Wang, Ruiming Han

**Affiliations:** 1 Institute of Water Ecology and Environment Research, Chinese Research Academy of Environmental Sciences, Beijing 100012, China; 222502020@njnu.edu.cn; 2 School of Environment, Nanjing Normal University, Nanjing 210023, China; 09386@njnu.edu.cn

**Keywords:** agricultural soil, potentially toxic elements (PTEs), principal component analysis (PCA), positive matrix factorization (PMF)

## Abstract

The increase in the concentration of potentially toxic elements in farmland soil attracts more and more attention. To identify the sources of potentially toxic elements in agricultural soils, 148 soil samples in Yingtan were selected as a case study, potentially toxic elements levels were analyzed, and principal component analysis (PCA) and positive matrix factorization (PMF) were employed. The results indicate that the average of Zn (89.62 mg·kg^−1^ d.w.), Cu (76.30 mg·kg^−1^ d.w.), Pb (35.56 mg·kg^−1^ d.w.), Mo (0.66 mg·kg^−1^ d.w.), and Cd (0.59 mg·kg^−1^ d.w.) exceed the corresponding soil background values of Jiangxi Province. Moreover, the high spatial coefficient of variation (above 1.00) for these elements suggests a significant influence from long-term external inputs. Among all of the elements above, the soil Cu and Cd concentrations indicate a relatively high pollution ranked by *Igeo*. Further analysis of sources apportioned by PCA and PMF implies that the potentially toxic elements input into agricultural soil may be attributed to mining activity, natural sources, smelting, and agricultural activity. This study implies that PCA-PMF combined with the field survey may be helpful tools for discerning the pollutants^’^ sources, and it addresses a view that the increasing Cu and Cd levels in farmland is concerning, as it is associated with the historical use of mixed fertilizers and a lack of supervision.

## 1. Introduction

Over the past four decades, highly intensive agricultural production alongside rapid industrialization and urbanization has led to increasingly severe farmland soil pollution and quality decline in China, posing a grave threat to agrarian product quality and safety [1]. According to the National Soil Pollution Status Survey Bulletin [2], 16.1% of the soil samples and 19.4% of the agricultural soils are contaminated based on China’s soil environmental quality limits, mainly by potentially toxic elements (PTEs), with cadmium pollution exceeding the standard the most severely (7.0%).

Currently, PTE pollution in China’s farmland is characterized by an expanding area and increasingly diverse sources [3]. Agricultural soil pollution distribution is spatially uneven, with contamination being more severe in the south and east compared to the north and west. Regions such as the Yangtze River Delta, Pearl River Delta, and the Northeast old industrial bases, which are major grain-producing areas with intensive industrial activities and high agricultural intensification, face prominent soil issues [4]. The sources of PTEs are also relatively complex, with parent materials and human activities being the main factors affecting their sources. Human activities mainly include industrial production emissions, sewage irrigation, mineral resource development, pesticide and fertilizer use, etc. [5,6]. However, with the modernization of agriculture and the development of the rural economy, the scope and severity of PTE pollution are expanding and worsening [7]. The safe utilization of agricultural soils in China is gathering increasing concern [8,9].

Exceeding the contaminant limits in food crops is widespread in some areas, especially Cd in rice in the southern region, which is often related to contaminated agricultural soils [10,11]. There are various inputs of contaminants, such as anthropogenic activities involving irrigation water, atmospheric deposition, utilizing fertilizers containing potentially toxic elements, accumulating hazardous waste containing PTEs [12,13], and even leachate from hazardous waste disposal [14,15,16]. Except for PTEs due to human activities, the background PTEs in soil or its acidic nature are thought to promote crop species or cultivars prone to PTE accumulation. An accurate identification of contaminant sources in agricultural systems is crucial for risk control strategies.

Source apportionment techniques can be categorized into qualitative source identification and quantitative source contribution. Qualitative methods primarily use principal component analysis (PCA) and isotope fingerprint analysis to identify major sources, while quantitative methods employ receptor models such as positive matrix factorization (PMF), geostatistical analysis, and chemical mass balance (CMB) [17,18,19,20]. PCA and PMF are widely used for PTE source apportionment in soils [21,22,23]. PCA simplifies data and reveals intrinsic structures by reducing dimensionality, which enhances source identification efficiency while preserving original data information. PMF allocates sources without prior knowledge of source profiles, which is advantageous when profiles are unknown or difficult to obtain [24]. However, PCA is qualitative, while PMF is quantitative, lacking an intuitive display of factor numbers and requiring multiple calculations [25]. Combining PCA and PMF compensates for their shortcomings, providing precise estimates of each element’s source contributions and minimizing biased judgments in source apportionment. For example, a study using this combined approach found that coal combustion (34.15%), livestock farming (17.44%), traffic emissions (12.42%), and natural factors (35.99%) were the main sources of pollution [26].

However, the complexity of the environment inevitably affects the accuracy of source resolution. If the sample number is limited, more variations may occur. Therefore, the combined auxiliary information should be obtained and integrated into PCA-PMF to improve the prediction’s accuracy.

The primary aim of this study is to develop PCA-PMF techniques by combined land-use type, which will be used for the source apportionment of PTEs in agricultural soils. This study emphasizes the advantage of the improved PCA-PMF and sheds light on the source of the PTEs of farmland, which is expected to help with risk reduction strategies on agricultural soil.

## 2. Materials and Method

### 2.1. Study Area and Sampling

**Figure 1:**
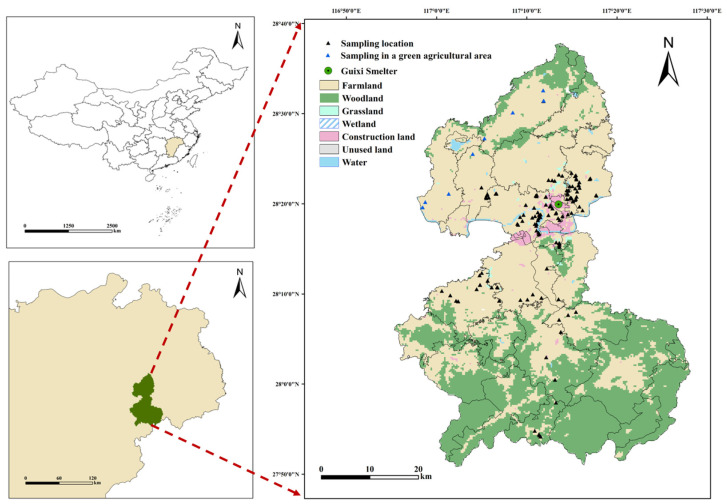
Sample sites.

The study area is located in Yingtan City, Jiangxi Province, China. It is characterized by a subtropical, humid monsoon climate; the region experiences high temperatures, ample sunlight, and abundant rainfall. The predominant soil type is Ferralsols (based on the FAO Soil Classification System), and the primary crop cultivated is rice. Yingtan City is endowed with abundant mineral resources, with reserves of Pb, Zn, Ag, and Mn ranking among the top in Jiangxi Province. The Guixi Smelter, the largest modern copper smelter in China, is located in the research area. Its main products include copper, sulfuric acid, electrolytic copper, electrolytic silver, etc. In 2022, a total of 148 agricultural soil samples (depth of 0–20 cm) were collected using clean stainless-steel shovels (Delixi Electric Co., Ltd., Leqing, China), each comprising a mixture of five closely located subsamples. After applying the quartering method, approximately 1 kg of the mixed soil sample was retained and stored in a polyethylene bag away from light, and the sampling information was recorded. The sampling sites are shown in Figure 1.

### 2.2. Field Investigation

Zhoufang Town (ZF Town) is situated at the northern end of Guixi City, Yingtan City, approximately 30 km from the Guixi Smelter. It is a typical green agricultural area with no industrial activities, sharing a similar climate, geological background, and agricultural cultivation situation with the aforementioned research area. According to the local data regarding the safe use and stringent control of contaminated agricultural land in 2021, certain rice samples have been identified as having PTE levels that exceed the limits set by the National Food Safety Standard (GB 2762-2012) [27].

Field investigations have been carried out along with face-to-face interviews to obtain more historical information on the farmland. The data reveal that the main irrigation water is taken from the local reservoir storage or a small river nearby. According to the villagers’ statements, it was confirmed that for some land, Cd rice exceeded the standard and had a history of mixed fertilizers being applied. The compound fertilizers had a lack of labels relating to PTE testing and were purchased from various sellers and producer sites, including Jiangxi, Hunan, Hubei, and Zhejiang. The usage of compound fertilizers is estimated to reach approximately 50~80% of the total fertilizer, but no detailed purchase and usage data have been recorded. Some sites are reported as old farmhouses or woodland; thus, they are assigned as soil without any fertilizer added. In some areas, slag piles in the surface soil have been observed along rural roads with low traffic volume, far away from rush-hour highways. The sample sites were classified based on the field investigation.

### 2.3. Analysis of PTEs

The soil samples were first sieved to remove debris such as roots, leaves, and stones. They were then dried at 60 °C to a constant weight for 48 h to ensure the removal of all free water. After drying, the samples were ground and passed through a 100-mesh sieve to obtain a homogeneous powder. All subsequent analyses were conducted on these prepared samples, and the results are reported on a dry weight basis (mg·kg^−1^ d.w.). The samples were comprehensively analyzed at the China Customs Science and Technology Research Center (STRC), which holds the necessary certification qualifications from China Metrology Accreditation (CMA) and is recognized as a proficiency testing provider by the China National Accreditation Service for Conformity Assessment (CNAS PT0012). The sample preparation and analysis process strictly adhered to the soil and sediment determination of aqua regia extracts of 12 metal elements by inductively coupled plasma mass spectrometry (HJ 803-2016) [28].

The determination of PTEs was carried out on an ICP-MS (PerkinElmer NexION 350X, Waltham, MA, USA) with a carrier gas flow rate of 1.10 L/min and a sampling depth of 6.9 mm. The instrument automatically measured three times. Two laboratory blank samples per batch were analyzed, with results below the detection limit. A zero-point analysis of the standard curve was conducted after each batch analysis. Furthermore, parallel sample analyses and spiked recovery tests were conducted on 10% of the samples per batch. The detection results met the standard requirements (refer to HJ 803-2016), with a relative standard deviation of less than 5% and a recovery rate ranging from 95.6% to 104.0%. A total of twelve PTEs were detected, including vanadium (V), chromium (Cr), manganese (Mn), cobalt (Co), nickel (Ni), copper (Cu), zinc (Zn), arsenic (As), molybdenum (Mo), cadmium (Cd), antimony (Sb), and lead (Pb).

### 2.4. Index of Geo-Accumulation

The Index of Geo-accumulation (*Igeo*), as introduced by Müller, is a widely recognized tool for assessing the degree of potentially toxic element contamination. It compares the element concentrations in the sampled soils to those in the background environment [29,30]. It is calculated using Equation (1).

(1) $$I_{geo}={log}_{2}\left[C_{n}/k\times B_{n}\right]$$

Here, $C_{n}$ (mg·kg^−1^ d.w.) represents the measured PTE content in sampled soil, while $B_{n}$ denotes the background value of potentially toxic elements in soil (mg·kg^−1^ d.w.) based on the soil background values in Jiangxi Province. k, usually denoted as 1.5, is the correction factor that indicates the possible variation of the background value of potentially toxic elements in the soil rock layer [30,31,32,33]. The *Igeo* values are categorized into seven classes, ranging from 0 to 6 (Table 1).

### 2.5. Principal Component Analysis (PCA)

The principal component analysis (PCA) adopted in this study is a dimensionality reduction method that applies a linear transformation to numerous interrelated original variables. It extracts fewer essential variables that are uncorrelated with each other. Using fewer representative factors, PCA can explain the primary information of multiple variables and infer relevant pollution sources, enabling the more accurate qualitative identification of pollution sources [34].

### 2.6. Positive Matrix Factorization (PMF)

The positive matrix factorization (PMF) employed in this study is a receptor model recommended by the US EPA and first introduced by Paatero and Tapper in 1994 [35]. This model identifies pollution sources using sample composition or fingerprints, applying non-negative constraints during the factor matrix decomposition process to achieve a quantitative analysis of pollution sources. In the PMF model, the concentration data matrix of receptor samples (X) is decomposed into a factor score matrix (G), a factor loading matrix (F), and a residual matrix (E). The matrix form is expressed as follows (Equations (2) and (3)):

(2) X=GF+E

(3) $$x_{ij}=\sum_{k=1}^{p}g_{ik}f_{kj}+e_{ij}$$

Here, $x_{ij}$ represents the concentration of compound *j* in sample *i* (mg·kg^−1^ d.w.); $g_{ik}$ denotes the contribution of source *k* to sample *i*; $f_{kj}$ is the concentration of compound *j* in source *k* (mg·kg^−1^ d.w.); p represents the total number of identified pollution sources in the model; and $e_{ij}$ is the residual for each sample and compound. The results of the PMF model are determined by the objective function (Equation (4)).

(4) $$Q\left(E\right)=\sum_{i=1}^{n}\sum_{j=1}^{m}{\left(\frac{e_{ij}}{u_{ij}}\right)}^{2}\;$$

Here, $u_{ij}$ represents the uncertainty of each compound in each sample, which is dimensionless, and the method for calculating uncertainty refers to the literature [36].

When the concentration of potentially toxic elements is below the detection limit of the corresponding method, the parameters are suggested as follows (Equation (5)):

(5) $$x_{ij}=\frac{d_{ij}}{2},\;u_{ij}=\frac{5}{6}d_{ij}$$

In reverse, the parameters can be set as follows. The value $x_{ij}$ can be seen as the PTE concentration detected. The uncertainty factor will be chosen depending on Equation (6) or (7).

(6) $$x_{ij}\leq {3d}_{ij},\;\;\;\;\;u_{ij}=\frac{d_{ij}}{3}+0.2\times c_{ij}$$

(7) $$x_{ij}>{3d}_{ij},\;\;\;\;\;u_{ij}=\frac{d_{ij}}{3}+0.1\times c_{ij}$$

Here, $c_{ij}$ is the measured concentration of the sample, mg·kg^−1^ d.w.; values of $x_{ij}$ are the input concentration during the model operation process, mg·kg^−1^ d.w.; $d_{ij}$ is the detection limit of the method, mg·kg^−1^ d.w.; and $u_{ij}$ is the uncertainty factor, dimensionless.

### 2.7. Data Analysis

The concentrations of each PTE were statistically analyzed using Microsoft Excel 2016. Subsequently, all test data were subjected to a normality test using the Kolmogorov–Smirnov test within SPSS 18.0 software, and the results were visualized using OriginPro 2024. The data are considered to have a normal distribution if the *p*-value is more significant than 0.05. For source apportionment analysis, the PCA module of SPSS 18.0 is employed in conjunction with PMF 5.0, downloaded from the US EPA website [37].

## 3. Results and Analysis

### 3.1. Descriptive Statistics of PTEs in Soil

The profiles of PTE levels in agricultural soil were surveyed (Table 2). The descending order of mean value in the soil is as follows: Mn, Zn, Cu, Pb, Cr, As, Ni, Sb, Mo, and Cd. It is noteworthy that the average concentrations of Mo and Pb are marginally elevated above the corresponding soil background values in Jiangxi Province. Also, the average of Cu and Cd are substantially higher, with 3.76 and 5.40 times the background levels, respectively. Of particular concern, over 80% of the surveyed sites had Cu and Cd concentrations surpassing the background values. Furthermore, compared to the risk screening values for soil contamination on agricultural land, as stipulated by the standard GB 15618-2018 [38], this indicates that the average of Cu and Cd are 1.53 and 1.97 times, respectively.

The coefficient of variation (CV) is defined as the degree of spatial variability in PTE concentrations [39], and it is sorted as low (CV ≤ 0.30), moderate (0.30 < CV ≤ 0.50), high (0.50 < CV ≤ 1.00), or extremely high (CV > 1.00). The CV values for PTEs in this study range from 0.51 to 2.56, with an average of 1.33 (Table 2), indicating an extremely high level of variation in the study area, which might be affected by external factors.

### 3.2. Assessment of Soil PTs Contamination

**Figure 2:**
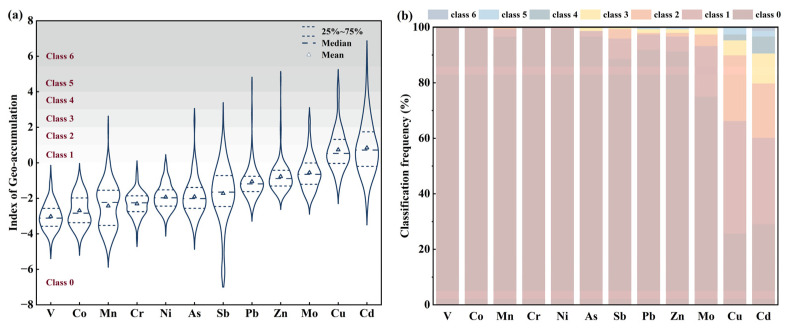
Index of Geo-accumulation in the study area.

To further investigate the degree of PTE pollution in agricultural soils, the Index of Geo-accumulation was applied. The regional *Igeo* mean is ranked from lowest to highest (Figure 2a), listed as V < Co < Mn < Cr < Ni < As < Sb < Pb < Zn < Mo < Cu < Cd. The pollution status of Cu and Cd in the soil is particularly concerning, with their respective *Igeo* mean values of 0.73 and 0.84, both falling within the class 1 zone (Table 1), which is a scope of moderate contamination. In contrast, all other PTEs fall in the zone of class 0, with no pollution. As far as how PTEs in soil fall into the scope of contamination is concerned, the *Igeo* indicates that Cu, Cd, Mo, and Sb are 74.32%, 70.95%, 25.00%, and 11.49%, respectively, whereas Mn, Ni, As, Pb, and Zn are below 10%. No contamination of V, Co, or Cr is found (Figure 2b). After calculating the *Igeo* value, it emphasizes the necessity to pay more attention to the risk that Cu and Cd may pose.

### 3.3. Source Apportionment of Soil PTEs

#### 3.3.1. Homology Analysis of Soil PTEs

The theory of elemental geochemistry puts up a view that the correlation among elements can reflect the similarities in their sources, and accordingly, the homogeneity analysis of PTEs can provide a basis for identifying the sources of PTEs [40,41,42], which is performed using correlation statistics and clustering analysis [40,43]. The content of soil PTEs in the study area was found to be normally distributed, determined by R-type clustering and Spearman correlation analysis.

**Figure 3:**
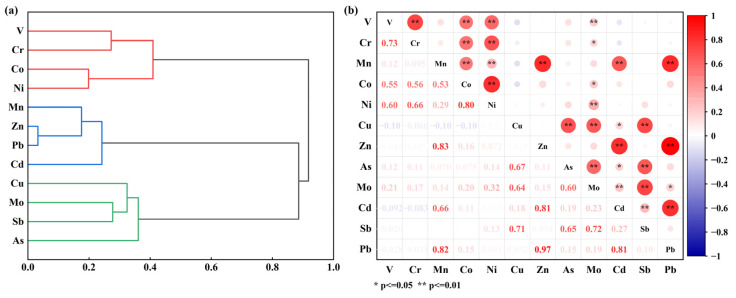
Homology analysis of potentially toxic elements in soil. (**a**,**b**) R-type cluster map and Spearman correlation heat map, respectively.

Figure 3a shows that PTEs are clustered into three types: Cluster I includes V, Cr, Co, and Ni; Cluster II includes Mn, Zn, Pb, and Cd; and Cluster III includes Cu, Mo, Sb, and As. The inter-cluster elements have significant autocorrelation, indicating that these PTEs may have homologous sources [44]. In conjunction with the correlation heat map (Figure 3b), it can be inferred that V, Cr, Co, and Ni in Cluster I may have homologous sources. Similarly, Mn, Zn, Pb, and Cd in Cluster II and Cu, Mo, Sb, and As in Cluster III are speculated as having homologous sources. A comparative analysis across the three clusters shows poor correlation among the elements in different clusters, suggesting a lower homology among these clusters. As a result, it can be concluded that there are at least three potential sources of PTEs in agricultural soils.

#### 3.3.2. PCA Analysis of Soil PTEs

**Figure 4:**
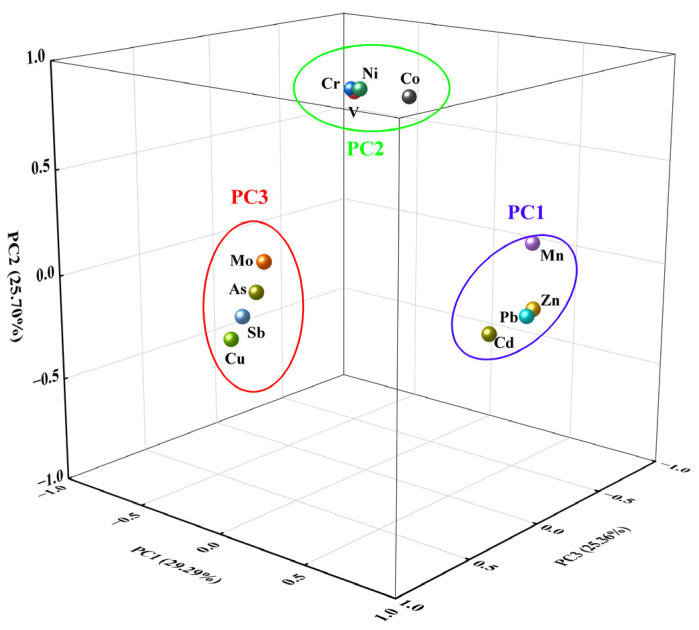
PCA loading contribution of potentially toxic elements.

PCA is further utilized to delineate the sources of PTEs in the soil. The Kaiser–Meyer–Olkin test gave a value of 0.76, a value suitable for factor analysis, and the Bartlett sphericity examination passed. Following varimax rotation to maximize variance, three principal components were identified, adhering to the criterion of eigenvalues exceeding 1 in PCA. These components cumulatively account for 80.34% of the total variance, suggesting that the majority of PTE sources can be attributed to these three principal components. The three factors after rotation are shown (Table 3), and the loadings on each factor are displayed (Figure 4).

The first principal component (PC1) explains 29.29% of the total variance, with high loadings (>0.85) observed for Mn, Zn, Cd, and Pb, which indicates that these elements likely share a homologous source or similar transport pathways, as evidenced by their significant correlations (Figure 3). Based on the Mineral Resource Master Plan of Yingtan City (2008–2015), it is confirmed that the area boasts a significant abundance of lead–zinc mineral resources. According to previous research, PTEs such as Pb, Zn, and Cd are released into the environment during the mining process and subsequently enter the soil through weathering, erosion, and other environmental processes [45,46,47]. Also, a large amount of wastewater and slag is discharged during the ore dressing process, which contains high concentrations of PTEs [46]. For example, in a typical lead–zinc mining area in Guizhou Province, the average concentrations of Pb and Zn in the ore dressing wastewater reach 10.5 mg/L and 25.3 mg/L, respectively, far exceeding the national discharge standards (GB 8978-1996) [48,49]. If these wastewaters are discharged directly without treatment, they can cause severe pollution to the soil and groundwater. The research area has a developed mining industry, which might be one of the pollution source contributors. Moreover, Cd has been identified as an element associated with agricultural practices, and the excessive use of phosphate fertilizers can result in Cd accumulation in soil [50,51]. Similar fertilizers were applied in the investigation areas; therefore, PC1 is assigned as a composite source encompassing both mining and agricultural activities.

The second principal component (PC2) explains 25.70% of the total variance, in which strongly positive correlations of V, Cr, Co, and Ni were explored, and the CV values of the four elements were all relatively weak (0.51~0.65); meanwhile, their average contents did not exceed the soil background values in Jiangxi Province. All the data suggest that external activities have minimal impact on their distribution. Concerning the source of V, Cr, Co, and Ni, the previous report indicated that natural sources originating from soil matrix matrices and weathering processes may be the main input [30,32,52,53]. Combined with the level of background PTEs in Jiangxi Province, it is speculated that PC2 may be a natural source.

The third principal component (PC3) explains 25.36% of the total variance, with Cu, As, Mo, and Sb being the main loading elements. Among them, Cu pollution is the most severe, with an average content exceeding 3.76 times the soil background value in Jiangxi Province. According to Zhou [54], the Guixi Smelter did not plan and control the discharge of waste residue, wastewater, and exhaust gas generated during the smelting process in the early stages of operation. After more than 30 years of accumulation, it has caused pollution to the surrounding environment, with the main pollutants including Cu. The investigation conducted by Wu et al. [55] and Xu et al. [56] also confirms this statement. In addition, substantial acid waste containing As, Mo, and Sb was generated from copper smelting and released into the environment by slag, wastewater, and flue gas [57,58]. These PTEs enter the soil via atmospheric deposition, waste residue leakage, sewage irrigation, and other pathways, resulting in enrichment in the soil. Thus, the main source of elements of PC3 can be identified as copper smelting activities.

#### 3.3.3. PMF Analysis of Soil PTEs

To further elucidate the origins and contributions of PTEs in agricultural soils, PMF was employed to determine the sources. The PTE concentration data and their corresponding uncertainty parameters were introduced into the PMF model. Based on the results from PCA, the parameters of three primary sources were set, but an unsatisfactory fitting was obtained. To improve the fitting degree, more source numbers were selected to reflect the multiple sources of soil PTEs in the research area, and the Robust model was adopted. The number of runs was set as 20, and the mode of “random starting seed number” was optioned in PMF, and then parameters of five source factors were identified. The fitting results between the real and predicted values are presented in Table 4. All PTEs exhibit high correlation coefficients (R²) above 0.50, indicating that the PMF simulation yields well-fitting results in response to the sources.

**Figure 5:**
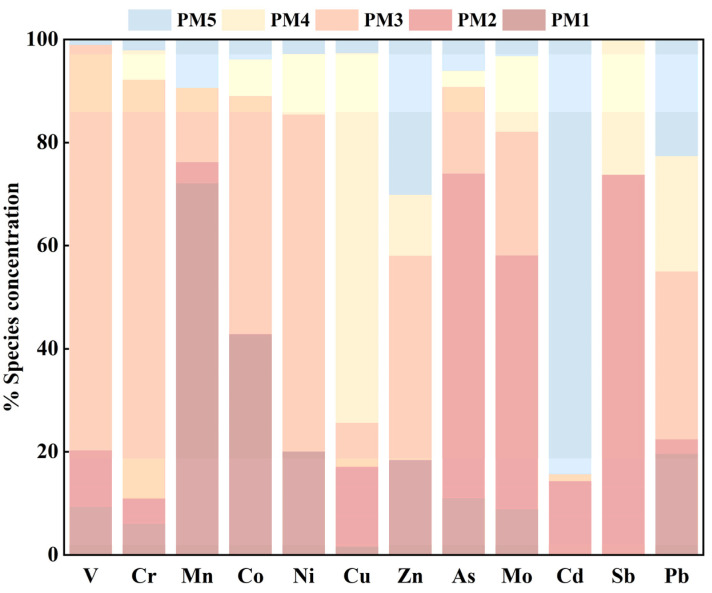
Factor fingerprints of potentially toxic elements.

In PMF, the loading factors for each potentially toxic element can help to apportion the sources (Figure 5), and it can be observed that PM1 is more loaded on Mn. However, the average concentration of Mn does not exceed the background values for Jiangxi Province, and only a few sample sites are found in a state of pollution. Regarding the sources, natural sources are the first focus. Mn, a constituent of the Earth’s crust, is naturally abundant in soils. Yingtan, a city with abundant manganese ore resources, is one of the main production areas of manganese ore in China. According to official statistical data, the manganese ore production in Yingtan has remained at more than 300,000 tons in the past few years. Most of these mines are open mines, and Mn would be released into the environment along with slag disposal and atmospheric sedimentation, which explains the increasing Mn soil in the local area. Considering the fact of mining activities, it is inferred that PM1 is mainly influenced by natural origin and mining activities.

The main loading factors of PM2 are Sb, As, and Mo, which overlap with the composition of PC3 in PCA. These elements are tightly associated with the by-products of copper smelting. Thus, PM2 is apportioned as an industrial source relating to copper smelting activities.

In PM3, the contribution ratios are dominated by V, Cr, Ni, Co, Zn, and Pb, with loading factor ratios of 78.67%, 81.14%, 65.41%, 46.12%, 39.58%, and 32.57%, respectively. It is worth noting that there is no correlation between the group of Zn and Pb and another group of V, Cr, Ni, and Co, which mainly come from soil parent materials and are less affected by human activities [30,32,52,53]. The study area was found to be rich in lead–zinc minerals and mining activities. In this study, the average contents of V, Cr, Ni, and Co are lower than the corresponding soil background values in Jiangxi Province (Table 2), with a low CV value, which means these PTEs come from natural sources, while soil Zn and Pb are contributed by anthropology activities. Therefore, PM3 can be characterized as a mixed source of natural origin and mining activities.

PM4 was mainly characterized by loadings from Cu. In this study, over 80% of the survey sites showed Cu concentrations exceeding the background value, with an average exceedance multiple of 3.76, which indicates that Cu has been related to severe external input. The copper smelting activities mentioned may have a role in contributing to Cu in soil. As far as other input ways, it cannot be ignored that Cu, as an inherent component of additives in livestock feed, will be transferred to the soil via animal manure [59]. In addition, using fertilizers and pesticides can lead to the accumulation of Cu in the soil [60]. So, PM4 is set as a mixed source of agriculture and copper smelting.

PM5 has a relatively high contribution of 84.37% to Cd. It was often regarded as a hallmark element in agricultural activities [50,51]. In general, some research found that phosphate ores contain 5–100 mg/kg of Cd, and most or all of the Cd enters the fertilizer [51]. Excessive application of phosphate fertilizer can cause the accumulation of Cd in the soil. Hence, PM5 is considered an agricultural source.

#### 3.3.4. Integrating PCA-PMF with Field Survey

**Figure 6:**
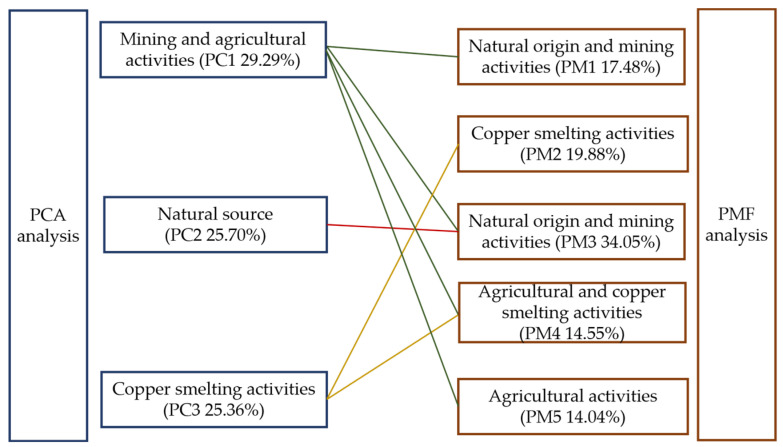
A comparison of source apportionment by PCA and PMF.

The source apportionment and counterpart contribution ratios obtained from both PCA and PMF are shown (Figure 6). PCA has accounted for the sources of PTEs in soil by using three factors, while PMF, based on three factors, has yielded suboptimal model fitting because of the complexity of input sources. Due to a complex environmental background, the pathways through which PTEs enter the soil are diverse, making it challenging to define them with a single source. The PMF model provides an advantage to overcome the shortage by optimizing the parameters of input sources and uncertainties. In terms of the source apportionment results, it can be observed that despite the use of five factors by PMF to describe the sources of PTEs, which still include the PCA analysis, the sources are mining activity, natural, smelting, and agricultural activity. This integration of PCA-PMF can be cross-validated for allocating pollution sources, which is more suitable for dealing with the complexity of PTE sources in soil.

The escalating pollution of agricultural soils with Cd and Cu not only impacts crop growth but also poses a prolonged risk to human health through the food chain [61,62]. This study underscores that the contamination of Cu and Cd in the study area’s soil warrants significant attention (Figure 2). For the specific area, an extra field survey combined with PCA-PMF can help apportion sources. For example, ZF Town, a green farmland area far away from the industrial zones, has found that Cd rice exceeded the standard. It is important to identify the source of PTEs. Information on historical land use in ZF is inquired, and the soil can be classified into two categories: soil with mixed fertilizer application (SF) and soil without mixed fertilizer application (SNF). The technology of PCA-PMF was employed in the specific green agricultural area, and the results are shown in Figure S2 and Table S4. The results show that Cd and Cu were alien pollution elements in farmland soils, and there was a significant correlation between them (Figure S1). In the previous analysis, it has been pointed out that soil Cd and Cu are closely related to the application of fertilizers. However, an interesting phenomenon was found in Table S1. The average of Cd in SNF soil is 0.06 mg·kg^−1^ d.w., which is considerably lower than the 0.18 mg·kg^−1^ d.w. observed in SF soil and the soil background value of 0.11 mg·kg^−1^ in Jiangxi Province. In contrast, the mean Cu concentration in SNF soil is 30.21 mg·kg^−1^ d.w., significantly lower than the 39.09 mg·kg^−1^ d.w. in SF soil, but it exceeds the background value of 20.3 mg·kg^−1^. This result indicates that fertilization is likely the predominant source of Cd, whereas Cu, although also present in higher concentrations in SF soil, may have additional anthropogenic sources contributing to its levels in the soil. For example, the long-term application of pig manure can lead to Cu accumulation in farmland soil [63,64]. This methodology of PCA-PMF merged with field survey will enable a more comprehensive understanding of the origins of Cd and Cu, particularly in the agricultural area.

## 4. Discussion

Cd and Cu contamination in farmland soil poses significant risks to human and environmental health. Cd, a toxic metal, enters the human body through the food chain, causing kidney damage, bone deformities (e.g., Itai-Itai disease), and increased cancer risk with long-term exposure [65]. It also impairs soil biota and plant growth, reducing biodiversity and ecosystem services [66]. Cu, essential in small amounts, becomes harmful at high levels, causing gastrointestinal, liver, and kidney issues in humans and disrupting the nervous system. In soil, elevated Cu levels inhibit microbial activity that is crucial for nutrient cycling and plant growth, leading to reduced crop yields and quality [67].

Given the complex pollution distribution in the study area, a multi-method approach is recommended for highly polluted small-area farmland patches. Deep plowing combined with guest-soil methods can dilute contaminants. Acidic modifiers like ferrous sulfate can adjust soil pH to precipitate Cd and Cu without harming the acidic environment. For bioremediation, local acid-tolerant hyperaccumulators such as Rumex species, which excel in Cd enrichment, can be planted in treated soils to boost phytoremediation efficiency. Additionally, acid-tolerant microorganisms like Acid thiobacillus can be introduced, which can transform Cd and Cu to enhance the removal of PTEs when cooperating with plants.

This highlights the need for government departments to enhance the environmental supervision of the copper-smelting industry, drive smelters to upgrade pollution-control facilities, ensure standard waste discharge, and curb PTE migration to farmland. They should strictly enforce ecological protection for mining, regulate mining procedures, improve drainage systems, and prevent PTE wastewater from entering farmland. Ecological restoration, like vegetation cover on abandoned mines, should be implemented to block PTE spread. In agriculture, scientific fertilization and pesticide use should be promoted. Organic fertilizer use should be encouraged to replace some chemical fertilizers, PTE input from fertilizer impurities should be reduced, and farmers should be guided on using low-toxicity, low-residue pesticides with minimal impact on soil PTEs, building a PTE source-control system.

Additionally, current research has limitations in scale, and relying solely on PCA and PMF may not fully capture the complexity of PTs sources, especially considering the influence of various environmental factors and the dynamic nature of soil interactions. It is recommended that introducing isotope methods could provide a more robust framework for source identification by offering geochemical fingerprints that are less affected by external conditions and more closely related to the compositional features of the source region.

## 5. Conclusions

This study suggests that the input of PTEs into agricultural soil may be attributed to mining activity, natural sources, copper smelting, and agricultural practices. Among these elements, Cu and Cd were significantly enriched in agricultural soils and identified as the most critical pollutants in the region. The comparative analysis using PCA and PMF demonstrated a high degree of congruence in identifying pollution sources, providing mutual validation for the large-scale region with complex sources. PCA excels in determining the primary number of predominant pollution sources, while PMF is more precise in calculating their contribution rates. For specific sites, the integration of PCA-PMF with field investigations effectively pinpoints the sources and has proven to be a robust approach in this study.

## Figures and Tables

**Table 1:** *I_geo_* of contamination degrees ranking.

Class	*I_geo_* Value	Soil Quality
0	*I_geo_* ≤ 0	Practically uncontaminated
1	0 < *I_geo_* ≤ 1	Uncontaminated to moderately contaminated
2	1 < *I_geo_* ≤ 2	Moderately contaminated
3	2 < *I_geo_* ≤ 3	Moderately to heavily contaminated
4	3 < *I_geo_* ≤ 4	Heavily contaminated
5	4 < *I_geo_* ≤ 5	Heavily to extremely contaminated
6	*I_geo_ >* 5	Extremely contaminated

**Table 2:** Statistical analysis of PTE concentrations in soil.

	Mean of All Soil ^(1)^ (mg·kg^−1^ d.w.)	Minimum ^(1)^ (mg·kg^−1^ d.w.)	Maximum ^(1)^ (mg·kg^−1^ d.w.)	Variation Coefficient	Soil Background Value in Jiangxi Province ^(2)^ (mg·kg^−1^)	Risk Screening Value ^(3)^ (mg·kg^−1^)	Average of the *Igeo*
Mn	137.88	14.63	1861.00	1.31	328.00	-	−2.43
Zn	89.62	25.81	2717.60	2.56	69.40	200.00	−0.77
Cu	76.30	10.23	739.05	1.39	20.30	50.00	0.73
Pb	35.56	7.47	990.83	2.48	32.30	80.00	−1.06
V	20.58	4.59	96.76	0.65	95.80	-	−3.02
Cr	15.47	3.49	57.14	0.51	45.90	250.00	−2.31
As	8.57	1.13	116.05	1.60	14.90	30.00	−1.91
Ni	8.42	2.13	28.55	0.57	18.90	60.00	−1.93
Co	3.21	0.66	11.36	0.65	11.50	-	−2.70
Sb	0.90	0.02	9.92	1.26	1.15	-	−1.73
Mo	0.66	0.16	4.19	0.98	0.50	-	−0.56
Cd	0.59	0.03	10.85	2.00	0.11	0.30	0.84

Notes: ^(1)^ The concentrations of the elements analyzed are reported on a dry weight basis (mg·kg^−1^ d.w.). ^(2)^ Soil background value of layer A in Jiangxi Province (background values of soil elements in China, China National Environmental Monitoring Centre, 1990). ^(3)^ Risk screening values for soil contamination of agricultural land, pH ≤ 5.5 (Soil Environmental Quality Risk Control Standard for Soil Contamination of Agricultural Land, GB15618-2018, Ministry of Ecology and Environment of China, 2018).

**Table 3:** Rotating loadings of potentially toxic elements on principal components.

Elements	Component		
	PC1	PC2	PC3
V	−0.06	**0.84**	0.02
Cr	−0.07	**0.85**	0.03
Mn	**0.89**	0.27	−0.06
Co	0.23	**0.85**	−0.01
Ni	0.07	**0.88**	0.14
Cu	−0.02	−0.13	**0.89**
Zn	**0.97**	0.01	0.03
As	0.08	0.09	**0.83**
Mo	0.13	0.24	**0.83**
Cd	**0.87**	−0.08	0.21
Sb	0.06	−0.01	**0.89**
Pb	**0.97**	−0.01	0.08
Eigenvalues	4.00	2.88	2.76
Variance contribution ratio %	29.29	25.70	25.36
Cumulative contribution ratio %	29.29	54.98	80.34

Note: (1) The rotation method used varimax with Kaiser Normalization, and the rotation converged in 4 iterations; (2) the bold numbers represent the higher values of the load in each principal component.

**Table 4:** Fitting results of measured value and simulated predicted value of potentially toxic element content.

Elements	R^2^	Intercept	Slope
V	0.68	6.92	0.60
Cr	0.85	1.82	0.85
Mn	0.64	58.86	0.47
Co	0.88	0.62	0.72
Ni	0.68	2.88	0.62
Cu	0.86	12.21	0.76
Zn	0.76	47.95	0.19
As	0.63	3.91	0.38
Mo	0.70	0.17	0.63
Cd	0.87	0.02	0.89
Sb	0.80	0.04	0.80
Pb	0.59	18.45	0.14

## Data Availability

The raw data supporting the conclusions of this article will be made available by the authors upon request.

## Supplementary Materials

The following supporting information can be downloaded at: https://www.mdpi.com/article/10.3390/toxics13040267/s1, Table S1: Statistical analysis of PTEs concentrations in the typical green agricultural soil; Table S2: Rotating loadings of PTEs on principal components; Table S3: Fitting results of measured value and simulated predicted value of PTEs content based on PMF; Table S4: Sources spectra and source contribution of PTEs based on PMF; Figure S1: The homology analysis of PTEs in the typical green agricultural soil; Figure S2: PCA loading contribution of the PTEs.
