# Supplementary material for: Source Apportionment of Potentially Toxic Elements in Agricultural Soils of Yingtan City, Jiangxi Province, China: A Principal Component Analysis–Positive Matrix Factorization Method
Source: Toxics. 2025 Mar 31;13(4):267. doi: 10.3390/toxics13040267 (PMC12030843; doi:10.3390/toxics13040267)
Supplement: Supplementary file 1 [file toxics-13-00267-s001.zip › toxics-3421931-supplementary.pdf]

Supplementary Information

Tables & Figures

Table S1 Statistical analysis of PTEs concentrations in the typical green agricultural soil.

	Mean of soil with mixed fertilizer application (SF) [1)] (mg·kg^{-1} d.w.)	Mean of soil without mixed fertilizer application (SNF) [1)] (mg·kg^{-1} d.w.)	Mean of all soil[1)] (mg·kg^{-1} d.w.)	Minimum[1)] (mg·kg^{-1} d.w.)	Maximum[1)] (mg·kg^{-1} d.w.)	Variation coefficient	Soil background value in Jiangxi Province[2)] (mg·kg^{-1})	The average of the *Igeo*
Mn	92.6	169.5	130.86	36.2	309.1	0.8	328	-2.27
Zn	49.83	59.83	54.54	31.7	96.18	0.43	69.4	-1.04
V	27.07	40.54	33.56	13.44	70.69	0.56	95.8	-2.29
**Cu**	**39.09**	**30.21**	**32.16**	**19.18**	**41.84**	**0.24**	**20.3**	**0.04**
Cr	23.96	26.66	24.87	10.94	45.83	0.46	45.9	-1.60
Pb	17.87	16.77	17.35	10.2	26.99	0.32	32.3	-1.55
Ni	8.9	14.18	11.45	4.33	25.07	0.68	18.9	-1.59
As	7.01	7.46	6.86	2.86	11.79	0.45	14.9	-1.85
Co	2.76	5.17	3.95	1.61	9.97	0.77	11.5	-2.45
Mo	0.42	0.6	0.55	0.22	0.89	0.48	0.5	-0.62
Sb	0.46	0.5	0.45	0.1	0.74	0.57	1.15	-2.24
**Cd**	**0.18**	**0.06**	**0.11**	**0.03**	**0.22**	**0.57**	**0.11**	**-0.76**

Notes: [1)] The concentrations of the elements analyzed are reported on a dry weight basis (mg·kg^{-1} d.w.). [2)] Soil background value of layer A in Jiangxi Province (Background values of soil elements in China, China National Environmental Monitoring Centre, 1990).

Table S2 Rotating loadings of PTEs on principal components.

Elements	Component	
	PC1	PC2
V	**0.94**	0.21
Cr	**0.97**	0.02
Mn	**0.81**	0.50
Co	**0.71**	0.56
Ni	**0.92**	0.37
Cu	0.17	**-0.92**
Zn	**0.92**	0.09
As	**0.91**	-0.21
Mo	**0.70**	0.56
Cd	-0.06	**-0.89**
Sb	**0.91**	-0.11
Pb	**0.75**	-0.03
Eigenvalues	7.82	2.36
Variance contribution ratio %	61.91	22.92
Cumulative contribution ratio %	61.91	84.83

Note: 1) The rotation method used varimax with Kaiser Normalization, and the rotation converged in 3 iterations; 2) The bold numbers represent the higher values of the load in each principal component.

Table S3. Fitting results of measured value and simulated predicted value of PTEs content based on PMF.

Elements	R^2	Intercept	Slope
V	0.924	5.696	0.759
Cr	0.930	3.765	0.818
Mn	0.989	-9.479	1.082
Co	0.792	0.868	0.702
Ni	0.995	0.734	0.914
Cu	0.604	-4.834	1.111
Zn	0.821	2.853	0.900
As	0.931	1.765	0.685
Mo	0.619	0.108	0.711
Cd	0.456	0.040	0.530
Sb	0.821	0.133	0.591
Pb	0.503	1.895	0.813

Table S4. Sources spectra and source contribution of PTEs based on PMF.

Elements	Source spectra ($mg \cdot kg^{-1}$)		Source contribution %	
	PM1	PM2	PM1	PM2
V	12.60	18.55	40.5	59.5
Cr	11.99	12.12	49.7	50.3
Mn	10.25	121.64	7.8	92.2
Co	0.80	2.83	22.1	77.9
Ni	2.67	8.52	23.9	76.1
Cu	25.06	5.85	81.1	18.9
Zn	22.43	29.47	43.2	56.8
As	3.95	2.52	61.1	38.9
Mo	0.20	0.30	40.8	59.2
Cd	0.10	0.00	99.9	0.1
Sb	0.19	0.21	47.3	52.7
Pb	8.10	7.91	50.6	49.4

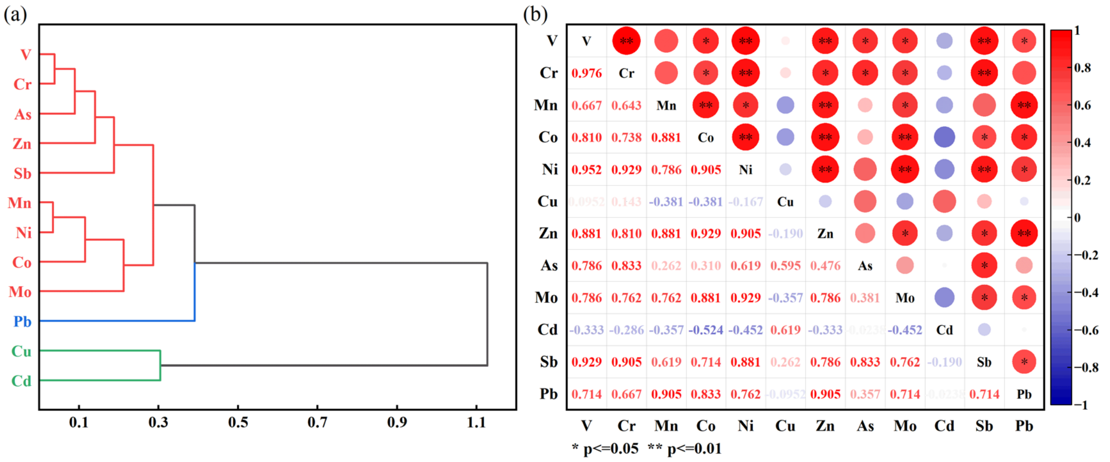

(a) and (b) are R-type cluster map and Spearman correlation heat map, respectively

Figure S1. The homology analysis of PTEs in the typical green agricultural soil.

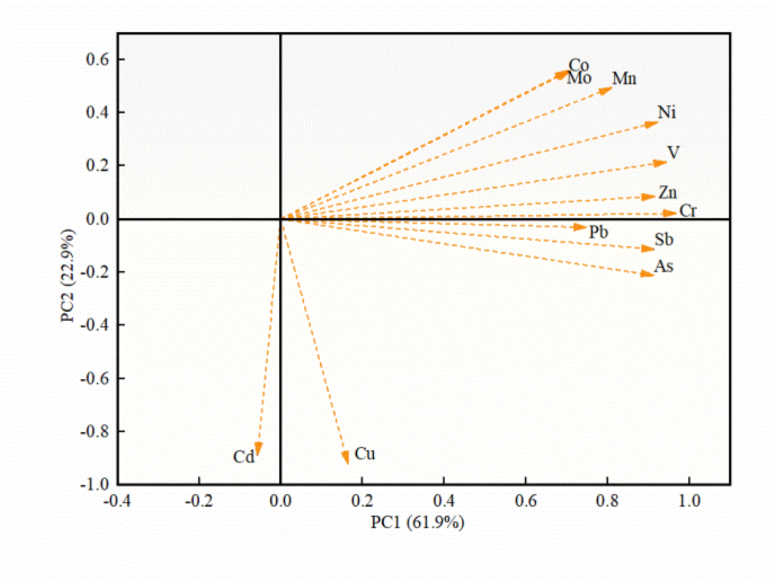

Figure S2. PCA loading contribution of the PTEs.
